# Human papillomavirus DNA as a factor determining the survival of bladder cancer patients.

**DOI:** 10.1038/bjc.1996.23

**Published:** 1996-01

**Authors:** A. Lopez-Beltran, A. L. Escudero, L. Vicioso, E. Muñoz, J. C. Carrasco

**Affiliations:** Department of Pathology, Cordoba University Medical School, Spain.

## Abstract

**Images:**


British Journal of Cancer (1996) 73, 124-127
© 1996 Stockton Press All rights reserved 0007-0920/96 $12.00

### Human papillomavirus DNA as a factor determining the survival of bladder cancer patients

A Lopez-Beltran[1], AL Escudero[1], L Vicioso[2], E Muñoz[3] and J Carlos Carrasco[4]

[1]*Department of Pathology, Cordoba University Medical School and Reina Sofia University Hospital, 14071 Cordoba, Spain;* [2]*Department of Pathology, Malaga University Medical School, 29071 Malaga, Spain; Departments of* [3]*Immunology and* [4]*Urology, Cordoba University Medical School and Reina Sofia University Hospital, 14071 Cordoba, Spain.*

**Summary** The natural history of transitional cell carcinoma (TCC) of the urinary bladder is somewhat variable, with a significant number of tumour recurrences that occasionally evolve towards an infiltrating disease. The aim of this study was to investigate the presence of human papillomavirus (HPV) DNA in 76 TCC specimens, and then correlate such findings with the overall patient survival. However, other classical prognostic clinical and pathological variables such as pathological grade and stage, koilocytosis, age and sex were also tested. HPV DNA was investigated by means of the highly sensitive polymerase chain reaction (PCR). DNA primers specific for HPV types 6, 11, 16 and 18 were used. Our results showed that 7 (9.21%) out of 76 such cases were reactive for HPV 16 DNA; one of them also reacted with HPV 6 DNA. The statistical analysis was done by the Kaplan–Meier method, Wilcoxon's generalised test for studying the differences in survival curves and Cox's regression analysis for independent prognostic factors. A significant *P*-value was found for pathological grade ($P<0.0001$) and stage ($P<0.0001$), HPV 16 DNA ($P=0.0418$) and koilocytosis ($P=0.0140$). Thus, pathological grade was the only independent factor in the bladder cancer survival. These observations may prove useful in prognostic stratification of patients with TCC of the bladder.

**Keywords:** human papillomavirus; polyamerase chain reaction; transitional cell tumour; urinary bladder

The presence of human papillomavirus (HPV) DNA has been reported most frequently in association with cervical dysplasias which can progress to malignancies, and benign condylomata acuminata (Stoler *et al.*, 1992; Donalson *et al.*, 1993). Recent studies indicate that some HPVs are associated with bladder carcinoma (Del Mistro *et al.*, 1988; Kitamura *et al.*, 1988; Querci Della Rovere *et al.*, 1988; Bryant *et al.*, 1991; Anwar *et al.*, 1992; Chetsanga *et al.*, 1992; Lopez-Beltran *et al.*, 1992*a*; Furihata *et al.*, 1993; Chang *et al.*, 1994; Lopez-Beltran and Muñoz, 1995). However, the exact incidence of HPV DNA involved in TCC of the bladder remains controversial (Chang *et al.*, 1994; Lopez-Beltran and Muñoz, 1995), since the reported incidence varies between 2.5% and 62% (Anwar *et al.*, 1992; Chetsanga *et al.*, 1992; Lopez-Beltran *et al.*, 1992*a*; Lopez-Beltran and Muñoz 1995). Negative results have been reported by Chang *et al.* (1994) and Ashfaq and Vuitch (1994). The prognostic implication of HPV infection in bladder cancer survival was suggested by Lopez-Beltran *et al.* (1992*a*) and Furihata *et al.* (1993), using non-isotopic DNA *in situ* hybridisation.

The aim of the present research was to investigate HPV incidence in 76 TCC specimens, using polymerase chain reaction (PCR) analysis, and then to correlate such findings with the overall patient survival. This study also included other classic prognosticators, such as the pathological grade and stage, patient age and sex, and koilocytosis. An attempt is also made to ascertain their possible prognostic implication in bladder cancer survival.

### Materials and methods

The study group consisted of 76 unselected and consecutive cases of TCC of the urinary bladder received at Reina Sofia University Hospital (Cordoba, Spain). All 76 patients underwent transurethral resection (TUR). Their mean age was $66.57 \pm 1.17$. Fourteen patients were female. Selected tissue specimens from all biopsies, formalin-fixed paraffin-embedded, were analysed for pathological grade and stage. All cases were followed over 5 years. Koilocytosis in TCC was evaluated following the criteria proposed by Hartveit *et al.* (1992).

Correspondence: A Lopez-Beltran, Unidad de Anatomia Patologica, Facultad de Medicina, Avda. Menendez Pidal s/n, 14071 Cordoba, Spain
Received 15 February 1995; revised 21 July 1995; accepted 9 August 1995

### *Sample preparation for PCR*

The paraffin-embedded tissues for PCR analysis were cut into 5–10 µm thin sections. To prevent contamination from one paraffin block to another, the knife and the microtome specimen holder were carefully cleaned with xylene after each specimen had been processed. To extract DNA, each section was placed into Eppendorf tubes and the paraffin removed twice with xylene and washed once with 0.5 ml of 100% ethanol to remove the solvent. The samples were then dried and resuspended in 300 µl of digestion buffer (50 mM Tris pH 8.5; 1 mM EDTA; 0.5% Tween 20 and 200 $\mu g\ ml^{-1}$ of proteinase K), and incubated for 6 h at 55°C. After this incubation the samples were heated at 95°C for 8 min to inactivate the proteinase K. DNA was then extracted twice with phenol–chloroform and the aqueous phase precipitated with 95% ethanol at −20°C overnight. After centrifugation at 13 000 r.p.m. for 15 min the DNA pellet was dried and dissolved in 50 µl of distilled water. The DNA was stored at −20°C until use.

### *PCR analysis*

PCR was performed mixing 10 µl of DNA with 90 µl of a solution containing 2 mM magnesium chloride, 50 mM potassium chloride, 10 mM Tris-HCl, pH 8.3, 0.01% gelatin, 200 µM dNTP, 2.5 U of *Taq* DNA polymerase and 2 µM of the following primers: for HPV type 6, the primers were HPV601 and HPV602, which amplify a region of 260 bp in the E5 gene. For HPV type 11 the primers were HPV114 and HPV115, which amplify a region of 350 bp in the L1 gene. For HPV type 16 and HPV type 18, the upstream primer was H1 which is common for both virus strains, and the downstream primers were H2 and H3 respectively, which amplify a 109 bp region from the open reading frame of the E6 gene (Table I). The primers were synthesised using an Applied Biosystem 381A DNA synthesiser (Foster City, CA, USA). The reaction was performed in an automated thermocycler

**Table I** Sequence of synthetic oligonucleotide primers and complementary oligonucleotide probes used for the PCR in this study

| *HPV type/gene* | *Primer or probe* | *Sequence (5′ to 3′)* | *Length (bp) of amplified products* |
|---|---|---|---|
| HPV 6/E5 | Primer HPV-601 | TAGTGGGCCTATGGCTCGTC | 260 |
| | Primer HPV-602 | TCCATTAGCCTCCACGGGTG | |
| | Probe HPV-603 | CATTAACGCAGGGGCGCCTGAAATTGTGCC | |
| HPV 11/L1 | Primer HPV-114 | GGAATACATGCGCCATGTGG | 350 |
| | Primer HPV-115 | CGAGCAGACGTCCGTCCTCG | |
| | Probe HPV-116 | CGCCTCCACCAAATGGTACACTGGAGG | |
| HPV 16/E6 | Primer H1 | ATTAGTGAGTATAGACATTA | 109 |
| | Primer H2 | GGCTTTTGACAGTTAATACA | |
| | Probe H4 | ATGGAACAACATTAGAACAGCAATACAACAAACCGTTGTG | |
| HPV 18/E6 | Primer H1 | ATTAGTGAGTATAGACATTA | 109 |
| | Primer H3 | GGTTTCTGGCACCGCAGGCA | |
| | Primer H5 | ATGGAGACACATTGGAAAAACTAACTAACACTGGGTTATA | |

**Table II** Selected variables representative of the 76 cases of TCC included in this study

| *Factors* | *Categories* | *No. deceased (%)* | *No. alive (%)* | *P-value* | *Overall no. (%)* |
|---|---|---|---|---|---|
| No. of patients | | 22 (100.0) | 54 (100.0) | | 76 (100.0) |
| Age[a] | Mean ± s.d. | 66.84 ± 2.45 | 66.88 ± 1.34 | | 66.57 ± 1.17 |
| Sex | Male | 18 (81.8) | 44 (81.4) | | 62 (81.6) |
| | Female | 4 (18.1) | 10 (18.5) | | 14 (18.4) |
| | | | | $P=0.8616$ | |
| Grade | I | – (0.0) | 14 (25.9) | | 14 (18.4) |
| | II | 4 (18.1) | 24 (44.4) | | 28 (36.8) |
| | III | 18 (81.8) | 16 (29.6) | | 34 (44.7) |
| | | | | $P=0.0001$[b,c] | |
| Stage | O | – (0.0) | 8 (14.8) | | 8 (10.5) |
| | A | 7 (31.8) | 38 (70.3) | | 45 (59.2) |
| | B | 11 (50.0) | 8 (14.8) | | 19 (25.0) |
| | C | 4 (18.1) | – (0.0) | | 4 (5.2) |
| | | | | $P=0.0001$[b] | |
| HPV 16 | + | 5 (22.7) | 2 (3.7) | | 7 (9.2) |
| | – | 17 (77.2) | 52 (96.2) | | 69 (90.7) |
| | | | | $P=0.0418$[b] | |
| Koilocytosis | + | 4 | 12 | | 16 (21.0) |
| | – | 18 | 42 | | 60 (79.0) |
| | | | | $P=0.0140$[b] | |

[a]Age at diagnosis (years). [b]Significant *P*-value. [c]Independent prognostic factor using Cox regression analysis.

(Perkin-Elmer, CT, USA) programmed for 40 cycles of DNA denaturation (95°C), primer annealing (65°C) and template extension (72°C). In the last cycle the extension step was 7 min. Amplified DNA fragments were hybridised with specific [$^{32}$P]ATP end-labelled probes. The complexes were electrophoresed in 12% SDS/PAGE gels, which were dried and exposed to XAR film for different periods of time. Primers PC03 and PC04 which amplified a 110 bp fragment of the human β-globin gene, were included as controls for the amount of DNA analysed. Necessary precautions to avoid cross-contamination were taken at all stages of extraction and amplification (Kwok and Higuchi, 1989).

### *Statistical analysis*

The statistical analysis was undertaken using the life test procedure. Univariate analysis of cancer-corrected 5 year survival (defined as death from or with bladder cancer) was done according to the Kaplan–Meier method (Kaplan and Meier, 1958). Differences between survival curves were estimated by the Wilcoxon test (Gehan, 1962). In addition, independent prognostic factors were sought by Cox regression analysis (Cox, 1972). A *P*-value below 0.05 was regarded as being statistically significant.

### Results

The selected variables representative of the 76 TCCs included in this study are illustrated in Table II. The PCR analysis showed positive signals for the HPV type 16 DNA in 7 (9.21%) out of 76 cases investigated. Likewise, one case showed reactivity for both HPV 16 DNA and HPV 6 DNA (Figure 1). Sixteen (21.0%) out of 76 cases had koilocytosis, and one of these HPV 16 DNA. Most patients with TCC (71.4%) associated with HPV DNA were of high pathological grade/stage, and died of disease within 9 to 13 months (Table III).

**Figure 1** Autoradiographs of positive signals for HPV DNA type 16 and HPV DNA type 6, using PCR analysis.

The univariate chi-squares for the Wilcoxon test showed pathological grade and stage, the presence of HPV 16-DNA and koilocytosis, to be significantly related with survival in all 76 cases. However, pathological grade was found to be an independent prognostic factor in patient survival (Cox regression analysis).

**Table III** Clinicopathological characteristics of the seven cases of TCC associated with HPV DNA

| *Case no.* | *Age* | *Sex* | *Follow-up (months)* | *Grade* | *Stage* | *PCR 16* | *Koilocytosis* |
|---|---|---|---|---|---|---|---|
| 14 | 64 | M | D/13 | III | B | + | – |
| 16 | 64 | M | D/10 | III | B | +[a] | – |
| 17 | 85 | M | D/10 | III | A | + | – |
| 22 | 60 | M | D/9 | III | B | + | – |
| 24 | 57 | F | NED/60 | I | O | + | + |
| 28 | 57 | F | D/13 | III | B | + | – |
| 30 | 51 | M | NED/60 | I | A | + | – |

[a]Reactive for both HPV DNA type 16 and HPV DNA type 6. D, death; NED, no evidence of disease; M, male; F, female.

**Table IV** Studies on HPV DNA of several types investigated in human urinary bladder carcinoma

| *Reference* | *Method/HPV type DNA studied* | *Prevalence no. (%)* | *HPV type DNA detected* |
|---|---|---|---|
| Kitamura *et al.* (1988) | SBH/HPV 16 | [a]1/10 (10.0) | HPV 16 |
| Querci *et al.* (1988) | SBH/HPV 11 | [a]1/1 (100.0) | HPV 11 |
| Bryant *et al.* (1991) | ISH/HPV 6/11, HPV 16/18 | 12/76 (15.7) | HPV 16/18 |
| Lopez-Beltran *et al.* (1992*a*) | ISH/HPV 6/11, HPV 16/18, HPV 31/33/35 | 9/18 (50.0) | HPV 16/18 |
| Chetsanga *et al.* (1992) | PCR/HPV 6, 11, 16, 18, 31, 33 | 1/44 (2.5) | HPV 16 |
| Anwar *et al.* (1992) | ISH/HPV 16 | 10/20(50.0) | HPV16 |
| | PRC/HPV 6, 11, 16, 18, 33 | 39/48 (81.0) | HPV 16, 18, 33 |
| Wilczynski *et al.* (1993) | SBH/PCR/HPV 6, 16, 18 | [b]1/22 (4.5) | HPV 6 |
| Furihata *et al.* (1993) | ISH/HPV 16,18, | 28/90 (31.1) | HPV 16, 18, 33 |
| Yu *et al.* (1993) | PCR/HPV | 28/53 (52.8) | HPV 16 |
| | | 2/53 (3.7) | HPV 18 |
| Salztein *et al.* (1993) | PCR | 0/33 (0.0) | HPV 6, 11, 16, 18, 31, 33 |
| Chang *et al.* (1994) | ISH/PCR/HPV 6, 11, 18, 31, 33, 35, 39, 40, 45, 51-59 | 0/108 (0.0) | - |
| Ashfaq *et al.* (1994) | ISH 6/11, 16/18, 31/33 | 0/8 (0.0)[c] | - |
| Lopez-Beltran *et al.* (1995) | ISH/HPV 6/11, 16/18, 31/33/33 | 4/76 (5.29) | HPV 16/18 |
| | PCR/HPV 6, 11, 16, 18 | 7/76 (9.2) | HPV 6, 16 |
| Current study | PCR/HPV 6, 11, 16, 18 | 7/76 (9.2) | HPV 16/6 |

[a] Patient with mild immunodeficiency. [b] Squamous cell carcinoma. [c] Two of eight reported cases were squamous cell carcinoma. SBH, Southern blot hybridisation; ISH, non-isotopic DNA *in situ* hybridisation.

### Discussion

Transitional cell carcinoma of the bladder is a heterogeneous group of neoplasms that typically present a variable biological potential including high risk of recurrence and frequent evolution towards an infiltrating disease with reduced survival rates (Lopez-Beltran *et al.*, 1994). The prognosis of bladder cancer seems to be related to pathological factors such as tumour grade and stage, although the immunohistochemistry of cell and tumour markers as well as flow cytometric analysis of abnormalities in DNA content have also been considered prognostically significant (Lopez-Beltran *et al.*, 1992*b*). The purpose of this paper was to determine whether or not the finding of HPV DNA in TCC has additional prognostic value in patient survival.

HPVs are known to infect man and although most of these proliferations are benign, some may become malignant, and this malignant transformation is related to HPV type (Howley, 1991). In the genitourinary tract, HPV types 6/11 are most commonly associated with genital condylomata acuminata (Del Mistro, 1988), whereas types 16 and 18 are associated with dysplasias and carcinomas (Chang, 1990). In TCC most HPVs were reported in a small number of patients with an immunodeficient status, (Kitamura, 1988; Querci Della Rovere, 1988). Although, recently a larger series of TCCs were screened, demonstrating a variable incidence of HPV DNA which ranged from 2.5% to 62% (Anwar *et al.*, 1992; Chetsanga *et al.*, 1992; Lopez-Beltran *et al.*, 1992*a*; Furihata *et al.*, 1993; Lopez-Beltran and Muñoz, 1995). Negative results were reported by Chang *et al.* (1994) and Ashaq and Vuitch (1994) (Table IV). In addition, HPV 16/18 DNA detected by means of non-isotopic *in situ* hybridisation has been related with a poor survival (Lopez-Beltran *et al.*, 1992*a*; Furihata *et al.*, 1993). Our results found a 9.2% incidence of HPV 16 DNA in TCC. Such differences could be explained by methodological reasons (Ashfaq and Vuitch, 1994). In fact, type and time of fixation have been considered important parameters for preservation of DNA (Greer *et al.*, 1991; Karlsen *et al.*, 1994). However, the finding presented here of a significant relationship between the detection of HPV 16 DNA and reduced patient survival, using PCR analysis confirmed previous reports on poor survival of TCC cases presenting with high-risk HPV DNA (Lopez-Beltran *et al.*, 1992*a*; Furihata *et al.*, 1993) detected by using *in situ* hybridisation. Taken together these results could indicate an additional prognostic value of viral infection in bladder cancer, although pathological grade is the only independent parameter in the survival of bladder cancer as showed by our results. This is in agreement with the finding that most patients with TCC (71.4%) associated with HPV 16 DNA were of high grade. Such results are of interest since pathological grade remains an important prognostic parameter in survival of patients with TCC of the urinary bladder. Finally, we found koilocytosis to be significant in patient survival, which could be related to the increasing incidence of koilocytosis concomitant with increasing pathological grade.

**Acknowledgement**
This research was supported by Fondo de Investigaciones Sauitarias (FIS) Grant 94/0064-01-02.

### References

ANWAR K, NAIKI H, NAKAKUKI K AND INUZUKA M. (1992). High frequency of human papillomavirus infection in carcinoma of the urinary bladder. *Cancer*, **70**, 1967–1973.

ASHFAQ R AND VUITCH F. (1994). Human papillomavirus and carcinomas of the female urethra. *J. Urol. Pathol.*, **2**, 195–201.

BRYANT P, DAVIS P AND WISON D. (1991). Detection of human papillomavirus DNA in cancer of the urinary bladder by *in situ* hybridization. *Br. J. Urol.*, **68**, 49–52.

CHANG F. (1990). Role of papillomavirus. *J. Clin. Pathol.*, **43**, 269–276.

CHANG F, LIPPONEN P, TERVAHAUTA A, SYRJÄNEN S AND SYRJÄNEN K. (1994). Transitional cell carcinoma of the bladder: failure to demonstrate human papillomavirus deoxyribonucleic acid by *in situ* hybridization and polymerase chain reaction. *J. Urol.*, **152**, 1429–1433.

CHETSANGA C, MALMSTRÖM PU, GYLLENSTEN U, MORENO-LOPEZ J, DINTER Z AND PETTERSON U. (1992). Low incidence of human papillomavirus type 16 DNA in bladder tumour detected by polymerase chain reaction. *Cancer*, **69**, 1208–1211.

COX DR. (1972). Regression models and life-tables. *J.R. Stat. Soc.*, **34**, 187–220.

DEL MISTRO A, KOSS LG, BRAUNSTEIN J, BENNETT B, SACCOMANO G AND SIMONS KM. (1988). Condylomata acuminata of the urinary bladder. Natural history, viral typing and DNA content. *Am. J. Surg. Pathol.*, **12**, 205–215.

DONALSON YK, ARENDS MJ, DUVALL E AND BIRD CC. (1993). PCR analysis of the upstream regulatory region of human papillomavirus genes in cervical intraepithelial neoplasia and cervical carcinoma. *J. Clin. Pathol.*, **46**, 1021–1023.

FURIHATA M, INOUE K, OHTSUKI Y, HASHIMOTO H, TERAO N AND FUJITA Y. (1993). High-risk human papillomavirus infections and overexpression of p53 protein as prognostic indicators in transitional cell carcinoma of the urinary bladder. *Cancer Res.*, **53**, 4823–4827.

GEHAN E. (1962). A generalized Wilcoxon test for comparing arbitrarily single-censored samples. *Biometrika*, **52**, 203–217.

GREER CE, LUND JK AND MANOS M. (1991). PCR amplification from paraffin-embedded tissues: Recommendations on fixatives for long-term storage and prospective studies. *PCR Methods and Applications*, **1**, 46–48.

HARVEIT F, MAELE BO AND THUNOLD S. (1992). Koilocytosis in neoplasia of the urinary bladder. *Br. J. Urol.*, **69**, 46–48.

HOWLEY PM. (1991). Role of human papillomavirus in human cancer. *Cancer Res.*, **51**, 5019s–5022s.

KAPLAN EL AND MEIER P. (1958). Non-parametric estimation from incomplete observation. *J. Am. Stat. Assoc.*, **53**, 457–481.

KARLSEN F, KALANTARI M, CHITEMERERE M, JOHANSSON B AND HARMAR B. (1994). Modifications of human and viral deoxyribonucleic acid by formaldehyde fixation. *Lab. Invest.*, **71**, 604–611.

KITAMURA T, YOGO Y, VEKI T, MURAKAMI S AND ASO Y. (1988). The presence of human papillomavirus type 16 genome in bladder carcinoma in situ of a patient with mild immunodeficiency. *Cancer Res.*, **48**, 7207–7211.

KWOK S AND HIGUCHI R. (1989). Avoiding false positives with PCR. *Nature*, **339**, 237–238.

LOPEZ-BELTRAN A AND MUÑOZ E. (1995). Transitional cell carcinoma of the bladder: Low incidence of human papillomavirus DNA detected by the polimerase chain reaction and *in situ* hybridization. *Histopathology*, **26**, 565–571.

LOPEZ-BELTRAN A, CARRASCO JC, REYMUNDO C, MORALES-JIMENEZ C, TORO-ROJAS M AND SANTAMARIA-OSSORIO M. (1992*a*). Bladder cancer survival and human papillomavirus infection. Immunohistochemistry and in situ hybridization. In *Oncogenes and Molecular Genetics of Urological Tumours*, Olsson, CA (ed.) pp. 83–89. Churchill Livingstone: Edinburgh.

LOPEZ-BELTRAN A, CROGHAN GA, CROGHAN I, HUBEN RP, METTLIN C AND GAETA JF. (1992*b*). Prognostic factors in survival of bladder cancer. *Cancer*, **70**, 799–807.

LOPEZ-BELTRAN A, CROGHAN GA, CROGHAN I, MATILLA A AND GAETA JF. (1994). Prognostic factors in bladder cancer. A pathologic, immunohistochemical, and DNA flow-cytometric study. *Am. J. Clin. Pathol.*, **102**, 109–114.

QUERCI DELLA ROVERE G, OLIVER RTD, MCMANCE DJ AND CASTRO JE. (1988). Development of bladder tumour containing HPV type 11 DNA after renal transplantation. *Br. J. Urol.*, **62**, 36–38.

SALZTEIN DR, ORIHUELA E, KOCUREK JN, PAYNE DA, CHAN TS AND TYRING SK. (1993). Failure of the polimerase chain reaction (PCR) to detect human papillomavirus (HPV) in transitional cell carcinoma of the bladder. *Anticancer Res.*, **13**, 423–425.

STOLER MH, RHODES CR, WITHBECK A, WOLINSKY SM, CHOW LT AND BROKER TR. (1992). Human papillomavirus type 16 and 18 gene expression in cervical neoplasia. *Hum. Pathol.*, **23**, 117–128.

WILCZYNSKI SP, OFT M, COOK N, LIAO SY AND IFTNER T. (1993). Human papillomavirus type 6 in squamous cell carcinoma of the bladder and cervix. *Hum. Pathol.*, **24**, 96–102.

YU ST, WU MM AND LI LM. (1993). Prevalence of human papillomavirus 16 and 18 in transitional cell carcinoma of the bladder. *Chin. Med. J.*, **106**, 494–496.


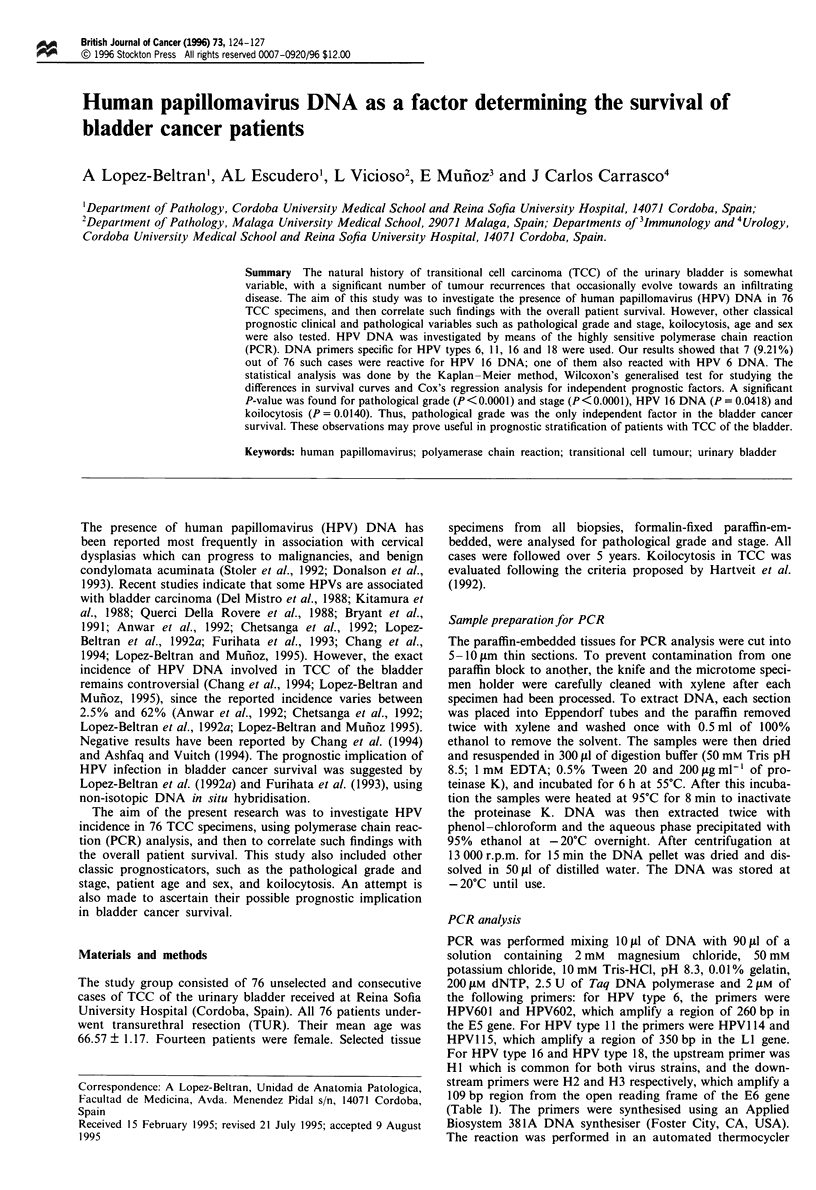

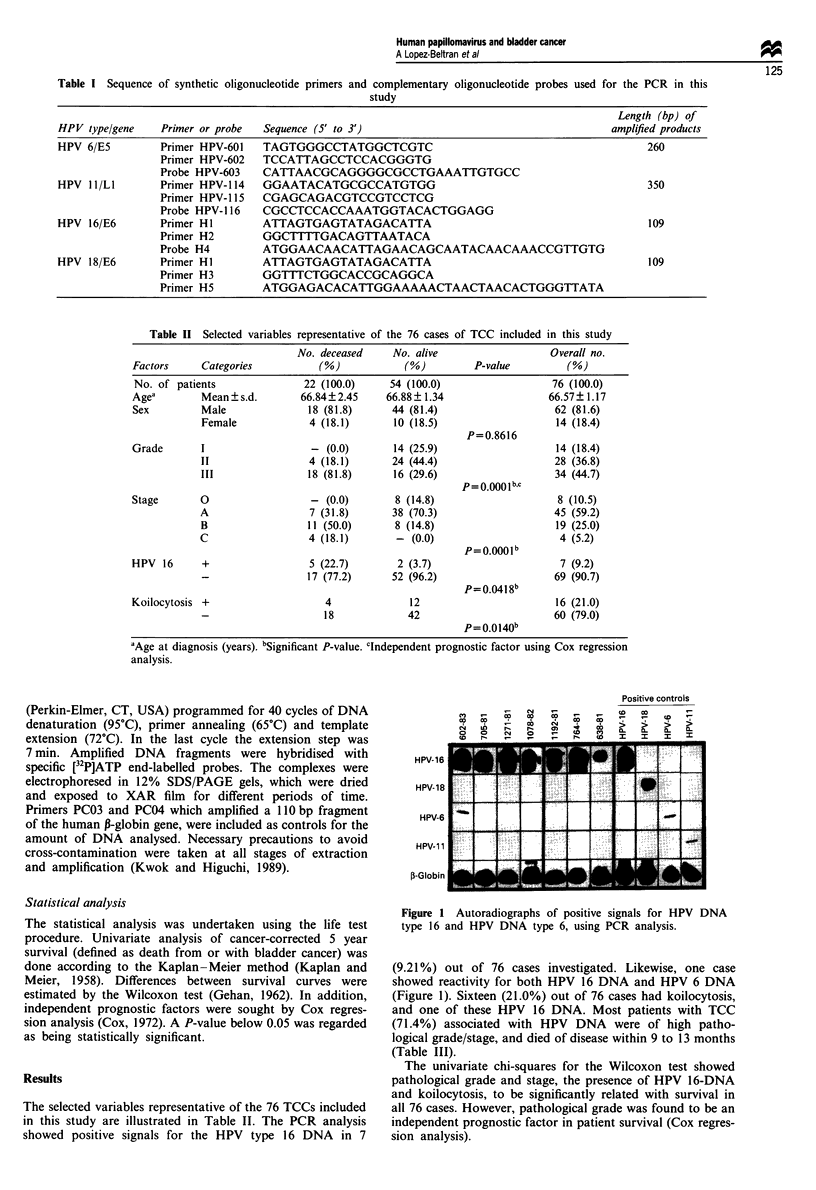

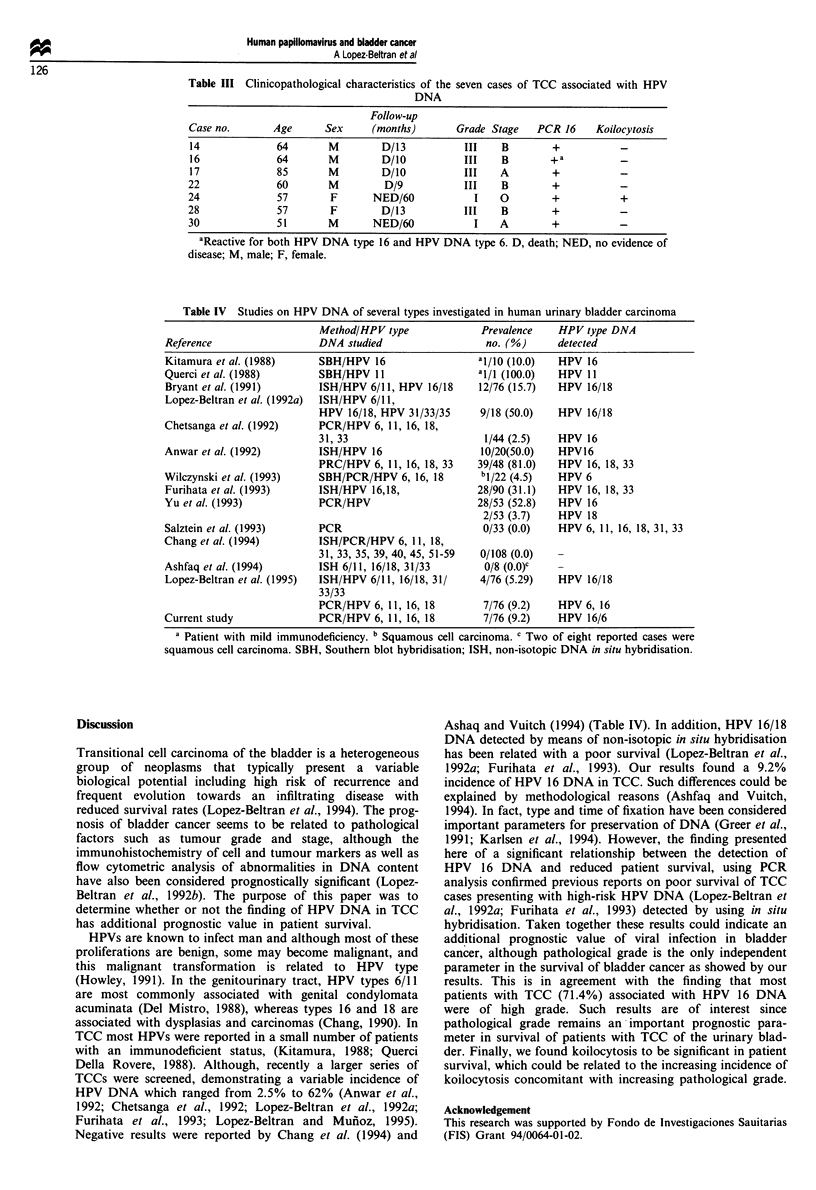

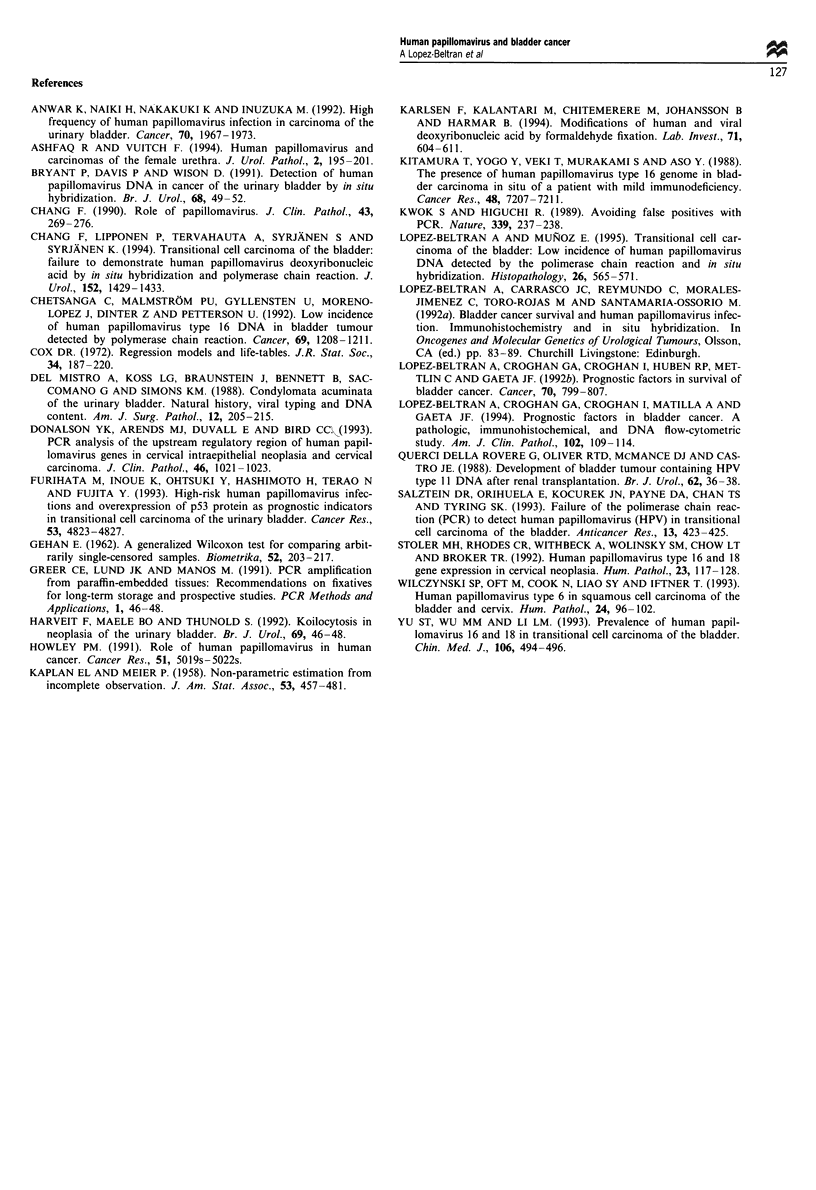


## References

[OCR_00527] Anwar K., Naiki H., Nakakuki K., Inuzuka M. (1992). High frequency of human papillomavirus infection in carcinoma of the urinary bladder.. Cancer.

[OCR_00533] Bryant P., Davies P., Wilson D. (1991). Detection of human papillomavirus DNA in cancer of the urinary bladder by in situ hybridisation.. Br J Urol.

[OCR_00542] Chang F., Lipponen P., Tervahauta A., Syrjänen S., Syrjänen K. (1994). Transitional cell carcinoma of the bladder: failure to demonstrate human papillomavirus deoxyribonucleic acid by in situ hybridization and polymerase chain reaction.. J Urol.

[OCR_00552] Chetsanga C., Malmström P. U., Gyllensten U., Moreno-Lopez J., Dinter Z., Pettersson U. (1992). Low incidence of human papillomavirus type 16 DNA in bladder tumor detected by the polymerase chain reaction.. Cancer.

[OCR_00561] Del Mistro A., Koss L. G., Braunstein J., Bennett B., Saccomano G., Simons K. M. (1988). Condylomata acuminata of the urinary bladder. Natural history, viral typing, and DNA content.. Am J Surg Pathol.

[OCR_00566] Donaldson Y. K., Arends M. J., Duvall E., Bird C. C. (1993). PCR analysis of the upstream regulatory region of human papillomavirus genomes in cervical intraepithelial neoplasia and cervical carcinoma.. J Clin Pathol.

[OCR_00573] Furihata M., Inoue K., Ohtsuki Y., Hashimoto H., Terao N., Fujita Y. (1993). High-risk human papillomavirus infections and overexpression of p53 protein as prognostic indicators in transitional cell carcinoma of the urinary bladder.. Cancer Res.

[OCR_00577] GEHAN E. A. (1965). A GENERALIZED WILCOXON TEST FOR COMPARING ARBITRARILY SINGLY-CENSORED SAMPLES.. Biometrika.

[OCR_00589] Hartveit F., Maehle B. O., Thunold S. (1992). Koilocytosis in neoplasia of the urinary bladder.. Br J Urol.

[OCR_00591] Howley P. M. (1991). Role of the human papillomaviruses in human cancer.. Cancer Res.

[OCR_00599] Karlsen F., Kalantari M., Chitemerere M., Johansson B., Hagmar B. (1994). Modifications of human and viral deoxyribonucleic acid by formaldehyde fixation.. Lab Invest.

[OCR_00605] Kitamura T., Yogo Y., Ueki T., Murakami S., Aso Y. (1988). Presence of human papillomavirus type 16 genome in bladder carcinoma in situ of a patient with mild immunodeficiency.. Cancer Res.

[OCR_00611] Kwok S., Higuchi R. (1989). Avoiding false positives with PCR.. Nature.

[OCR_00632] Lopez-Beltran A., Croghan G. A., Croghan I., Huben R. P., Mettlin C., Gaeta J. F. (1992). Prognostic factors in survival of bladder cancer.. Cancer.

[OCR_00634] Lopez-Beltran A., Croghan G. A., Croghan I., Matilla A., Gaeta J. F. (1994). Prognostic factors in bladder cancer. A pathologic, immunohistochemical, and DNA flow-cytometric study.. Am J Clin Pathol.

[OCR_00615] Lopez-Beltran A., Muñoz E. (1995). Transitional cell carcinoma of the bladder: low incidence of human papillomavirus DNA detected by the polymerase chain reaction and in situ hybridization.. Histopathology.

[OCR_00640] Querci della Rovere G., Oliver R. T., McCance D. J., Castro J. E. (1988). Development of bladder tumour containing HPV type 11 DNA after renal transplantation.. Br J Urol.

[OCR_00647] Saltzstein D. R., Orihuela E., Kocurek J. N., Payne D. A., Chan T. S., Tyring S. K. (1993). Failure of the polymerase chain reaction (PCR) to detect human papilloma virus (HPV) in transitional cell carcinoma of the bladder.. Anticancer Res.

[OCR_00650] Stoler M. H., Rhodes C. R., Whitbeck A., Wolinsky S. M., Chow L. T., Broker T. R. (1992). Human papillomavirus type 16 and 18 gene expression in cervical neoplasias.. Hum Pathol.

[OCR_00654] Wilczynski S. P., Oft M., Cook N., Liao S. Y., Iftner T. (1993). Human papillomavirus type 6 in squamous cell carcinoma of the bladder and cervix.. Hum Pathol.

[OCR_00659] Yu S. T., Wu M. M., Li L. M. (1993). Prevalence of human papillomaviruses 16 and 18 in transitional cell carcinoma of bladder.. Chin Med J (Engl).

